# Bacterial load assessment and multi-drug resistant Bacteria isolation from Fuchka in Mymensingh City, Bangladesh

**DOI:** 10.1016/j.onehlt.2025.101170

**Published:** 2025-08-18

**Authors:** Bushra Benta Rahman Prapti, Md. Tanjir Ahmmed, Nishita Ghosh Proma, Durratul Zanan Aunu, Shumia Islam Shampa, Aminur Rahman, Md. Shafiqul Islam, Mahbubul Pratik Siddique

**Affiliations:** a Department of Microbiology and Hygiene, Faculty of Veterinary Science, Bangladesh Agricultural University, Mymensingh 2202, Bangladesh; b Department of Food Engineering and Technology, Faculty of Agricultural Engineering and Technology, Bangladesh Agricultural University, Mymensingh 2202, Bangladesh

**Keywords:** Fuchka, Food-borne bacteria, Antibiogram, Multi-drug resistant, One health

## Abstract

Fuchka, a popular street food across Bangladesh and the Indian subcontinent, poses potential public health risk due to substandard hygienic practice during preparation and handling. This study evaluated the microbiological quality of *fuchka* and its associated components and identified food-borne bacteria with their antibiogram profiles within a One Health framework. 60 samples including fuchka, salad, hand wash, and dish wash water were collected from street vendors, shop, and restaurants within the Mymensingh City Corporation area (4 samples × 15 sites). Microbiological assessments included total viable count, total coliform count, and total staphylococcal count. Isolates were identified using polymerase chain reaction (PCR) and matrix-assisted laser desorption ionization time of flight mass spectrometry (MALDI-ToF-MS), and antibiotic susceptibility profiles were determined. The highest bacterial load was found in shop-based *fuchka* samples (10.5 ± 4.7 log CFU/g) while the lowest was in restaurant hand wash. Statistically significant differences were observed in TCC and TSC (*p* ≤ 0.05) across categories, with restaurant samples showing the lowest levels. In total 123 bacterial isolates were recovered, predominately *Klebsiella pneumoniae* (24.4 %), followed by *Staphylococcus* spp., *Escherichia coli*, *Enterobacter* spp., and *Citrobacter freundii*. Resistance to amoxicillin was observed in 100 % of Gram-negative isolates. Multidrug resistance was identified in 90 % of *K. pneumoniae*, 57 % of *E. coli*, 50 % of *Staphylococcus* spp., and 8.7 % of *Enterobacter* spp., with no MDR detected in *C. freundii*. The MAR index ranged from 0.1 to 0.6. These findings highlight urgent public health concerns and emphasize the need for integrated food safety strategies under the One Health approach.

## Highlights

• First detailed microbiological profiling of fuchka, in Bangladesh.
• First time PCR, MALDI-ToF-MS and antibiogram of bacteria of fuchka.
• Highest microbial load in shop-based vendors, while lowest in restaurant source.
• Frequently isolated bacteria included *K. pneumoniae*, *Staphylococcus* spp., *E. coli.*

## 1 Introduction

Various ready-to-eat (RTE) foods, including beverages and homemade cuisines, are classified as street foods, typically sold by vendors in public spaces and consumed readily without further preparation [1]. Approximately 2.5 million people consume various street foods, either from vendor origins or restaurants, because they are affordable and easily accessible [2]. Globalisation, urbanisation, and intercultural interaction have had a combined impact on substantially altering dietary habits, particularly in urban populations [3]. About 128 varieties of street food are sold daily, including fuchka (Phooska, Fuska, panipuri), spicy puffed rice (jhalmuri), bhelpuri, chickpea masala (chhola), spicy onion fritters (pianju), singara, and somucha in metropolitan areas like Dhaka, [4].

Fuchka is widely recognised as the most popular street food [5] amongst people of all classes and ages [6] in Bangladesh and the Indian subcontinent. Fuchka has various names depending on the regions: Gol Gappa in Delhi, Phuchka in West Bengal and Bangladesh, and pani puri in Nepal [7]. Even though highly popular, f*uchka* poses noteworthy food safety concerns due to its perishable nature and the fact that it is prepared, handled, and served without adequate hygiene [3].

Globally, foodborne illness outbreaks, caused by a diverse group of pathogens, exhibit both endemic and epidemic characteristics and have become a pressing public health issue that necessitates immediate attention [8]. Even in developed countries, nearly one-third of the population suffers from foodborne illnesses yearly [9]. In the United States, from 2000 to 2010, about 47.8 million cases of foodborne illness were reported, with 9.4 million cases associated with identifiable pathogens [10]. In developing countries, foodborne and waterborne diseases are serious health issues [9]. For example, Feglo and Sakyi [11] detected microorganisms, including *Staphylococcus aureus, Bacillus, Klebsiella pneumoniae,* and *E. coli* in the foods of street vendors in Ghana. Between 2003 and 2017, China experienced 19,517 foodborne disease outbreaks, resulting in 0.24 million illnesses, 0.11 million hospitalisations, and 1457 deaths. More importantly, amongst the pathogen-identified 13,307 outbreaks, 6.8 % were caused by *Salmonella*, 4.2 % by *Staphylococcus aureus*, and 3.0 % by *Bacillus cereus* [12].

In low- and middle-income countries, such as Bangladesh, where inferior food hygiene practices are often prevalent at street food vending sites, the presence of pathogenic bacteria at levels above acceptable limits poses a serious public health risk, because RTE foods are consumed without additional treatment [13]. Annually, approximately 30 million people in Bangladesh suffer from food and waterborne illnesses [3]. Studies have identified harmful microorganisms in various ready-to-eat foods. These microorganisms comprise *Escherichia coli, Enterobacter sakazaki, Citrobacter freundii, Salmonella typhimurium,* and *Vibrio cholerae* in Dhaka city [14]. Moreover, *Escherichia coli, Staphylococcus, Bacillus, Kurthia, Planococcus*, *Micrococcus*, *Listeria*, *Renibacterium, Klebsiella pneumoniae, Yersinia pestis*, *Enterobacter aerogenes,* and *Plesiomonas shigelloides* have also been detected in the Dinajpur district [6,14]*.*

Furthermore, the survivability and transmission dynamics of antimicrobial-resistant microorganisms have hampered food safety worldwide [3,13]. To this end, the association between RTE food consumption and the spread of multidrug-resistant (MDR) pathogens has already been revealed [15]. Fuchka's main ingredients are of animal origin, including eggs, yoghurt, and chutney, and these ingredients may harbour antibiotic residues and disseminate MDR pathogenic bacteria [16]. The utilisation of antimicrobials in the agricultural and veterinary sectors (for prophylactic, therapeutic, and metaphylactic purposes) has notably contributed to the development of multidrug-resistant (MDR) pathogens and antibiotic residues in animal foods [17]. Moreover, fuchka's preparation and sale often occur under unhygienic conditions with multiple contamination sources, including raw ingredients, water, vendor handling, and environmental exposure. These factors collectively render fuchka an ideal indicator for determining microbial contamination and AMR risks in street foods [13,15].

Rising concerns exist over food safety and antimicrobial resistance (AMR) in street foods, such as fuchka, accentuating the need for a more comprehensive understanding of the problem. However, these issues cannot be addressed through a singular intervention. Instead, they display interconnected challenges of human health, food production systems, and environmental hygiene. Therefore, a holistic and integrated approach directed by FAO-OIE-WHO under the ‘One Health Umbrella’ must be adopted and pursued to combat this situation [18]. Issues including the indiscriminate use of antibiotics, inadequate knowledge of antibiotic uses, ignorance, deficiencies in laws and standards for antibiotic production and sale, intensive animal rearing and aquaculture practices, and similar factors have aggravated the AMR situation, making it a major global concern over the last two decades [19,20]. Responding to growing concerns, recent One Health policies endorse coordinated, cross-sectoral actions to ensure food safety and reduce AMR transmission for sustainable human health and environmental sectors. These policies encompass the precise use of antibiotics, the appropriate handling of food to prevent cross-contamination, and hygienic environmental conditions [17].

Studies on Bangladesh's street foods have addressed issues, including bacteriological quality, bacterial isolation, and antibiotic resistance, even though few have highlighted fuchka [3,21,22]. Nonetheless, these works underscore the typical condition of street-vended foods. Some studies comprised fuchka [5,6,16,23], whereas Hossain and Habib [21] emphasised hygienic awareness amongst fuchka vendors and consumers. Hasan et al. [2] detected drug-*resistant E. coli* and *Staphylococcus* spp. in fuchka samples from Mymensingh city. Fuchka's food safety issues require special attention because it is highly popular, has multiple exposure routes and poses associated risks to human and environmental health. Hence, the objective of this study was to systematically determine the microbiological safety of fuchka and its components, and to contribute to the One Health initiative aimed at mitigating foodborne illness and antimicrobial resistance.

## 2 Materials and methods

### 2.1 Sampling areas and collection of fuchka samples

**Fig. 1:**
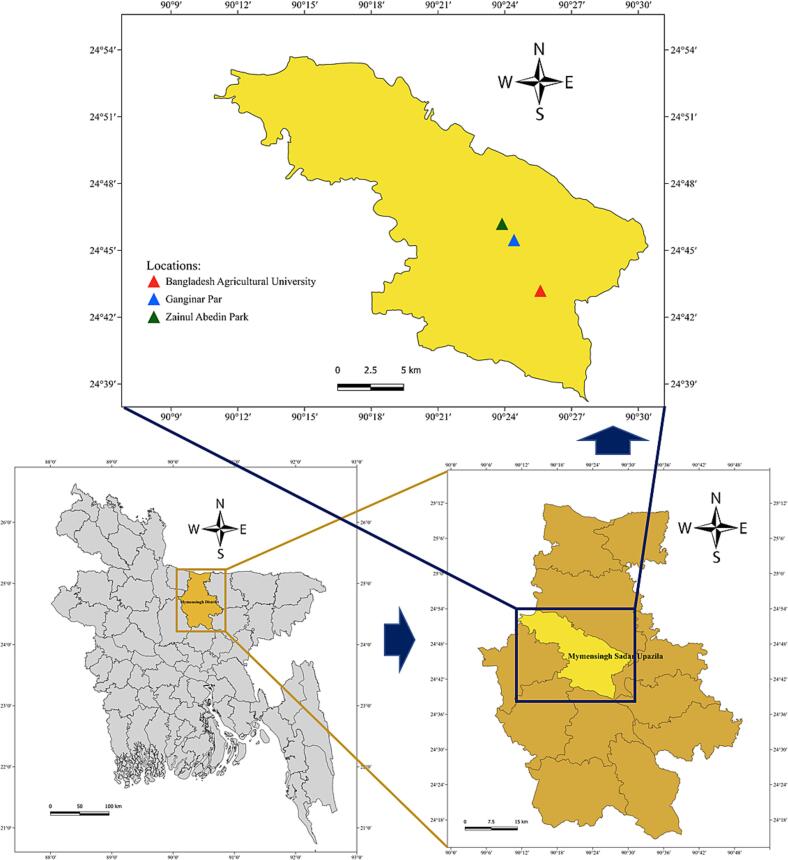
Map showing sampling locations within Mymensingh City Corporation area. The map was generated using QGIS software (version 3.36.1).

For this study, 60 samples were collected from various locations within the Mymensingh city corporation area (Fig. 1). These samples comprised crispy fuchka balls with filling and toppings (chickpeas, mashed potatoes, and sour yoghurt), salad (cucumber, tomato, onion, coriander leaves, and shredded eggs), dishwashing water (samples collected from the water used by vendors to clean utensils), and sellers' hand wash water (obtained by rinsing the hands of food vendors with sterile buffered peptone water) (Table S1). Sampling areas were categorised as follows: street-vended fuchka selling points, fuchka selling shops, and restaurants; based on physical setup, food handling practice and hygiene condition. Detail characteristics of each category are presented in Table 1 to provide a clearer understanding of the sampling context. The solid samples (fuchka and salad) were collected in sterile zip-lock bags. Conversely, liquid samples (dishwashing water and hand wash water) were collected in both zip-lock bags and 50-mL sterile Falcon tubes. All samples were labelled correctly and readily transported in an icebox to the Food Hygiene Laboratory, ensuring that the sample integrity was preserved. Before collection of samples from each selling point, the verbal consent from the shop owner or restaurant owner was taken, though a standard owner consent form was prepared. The whole experiment was ethically approved by the Experimentation Ethics Committee of the Bangladesh Agricultural University, Mymensingh [Approval No.: AWEEC/BAU/2023(46), Dated: 13.11.2023].

**Table 1:** Comparative characteristics of fuchka selling points included in the study.

Fuchka selling points types	Physical setup	Food handling practices	Hygiene conditions observed
Fuchka shops	Permanent or semi- permanent enclosed shop with seating area for consumer; fixed location	Preparation and serving from behind a counter, usually beside the seating area; utensils washed on-site (used municipal tap water or utensils rinsed in a bucket); occasional use of gloves; food stored in covered containers	Access to running water; presence of basic hand washing facility, moderately clean preparation surface; waste disposed in nearby bin
Roadside vendors	Mobile pushcart or tricycle with open-air setup; semi-fixed location near markets or roads	Food prepared on cart; utensils sometimes reused without thorough washing; minimal use of gloves; food often left uncovered during peak hours	No direct water supply; cleaning water reused multiple times; surfaces visibly soiled; waste scattered nearby
Restaurants	Permanent enclosed building with indoor seating; fully fixed location	Food prepared in a designated kitchen area; utensils washed with running water; gloves or serving spoons more commonly used; food stored covered	Access to running water and soap; dedicated washing sinks; relatively clean preparation and serving areas; organized waste disposal

### 2.2 Sample processing

Each fuchka sample (15 g) was ground and homogenised with 135 mL of sterile PBS with a stomacher blender (Stomacher Homogeniser, BioBase Biodustry Limited, Shandong, China) and thoroughly mixed. Similarly, salad samples (5 g) were minced, ground, and homogenised with 45 mL of sterile PBS. Then, the homogenised samples were subjected to a 10-fold serial dilution for microbiological analysis to determine the total viable count (TVC), total coliform count (TCC), and total staphylococcal count (TSC).

### 2.3 Bacterial load assessment by determination of TVC, TCC, and TSC

The Plate Count Agar (PCA), Eosin-methylene blue (EMB) agar, and Mannitol Salt agar (MSA) were utilised to enumerate TVC, TCC, and TSC, respectively [25]. A 50 μL of diluted food samples from each dilution was plated and spread with a disposable sterile L-shaped plastic bacterial cell spreader (agar plate spreader, Hunan BKMAM International Trade Co. Ltd., Hunan, China). Plates were dried and sealed with Parafilm (Bemis, USA), and then incubated overnight at 37 °C. Later, colonies (30−300) were counted, and CFU/g was determined by multiplying the average colony number by the dilution factor, as Sieuwerts et al. [25] described.

### 2.4 Isolation of contaminating bacteria

The aerobic bacteria were isolated using the method described by Patra et al. [26]. Each sample was enriched in nutrient broth (HiMedia, India) and incubated for 24 h at 37 °C. Pure culture was obtained through repeated sub-culturing on various selective and differential bacteriological culture media with the streak plate method. The culture media consisted of MacConkey agar, Eosin Methylene Blue (EMB) agar, Salmonella-Shigella (SS) agar, Xylose Lysine Deoxycholate (XLD) agar, Mannitol Salt Agar (MSA), and HiCrome UTI agar (HiMedia, India) [26].

### 2.5 Identification and biochemical characterisation of isolated bacteria

Bacteria were identified using the standard protocol of the International Commission on Microbiological Specifications for Foods (ICMSF) [27]. Colony characteristics, Gram's staining method, and routine biochemical tests (basic five-sugar fermentation tests, Methyl Red (MR), Voges-Proskauer (VP), the indole test, the oxidase, and catalase test) were conducted based on the standard methods specified by Patra et al. [26].

### 2.6 Extraction of genomic DNA of the bacterial isolates

The boiling method was employed to extract genomic DNA of the bacterial isolates. Briefly, 1 mL of seeded broth was centrifuged at 5000 rpm for 3 min. Then, the supernatant was discarded, and 200 μL of distilled water was added to resuspend the pellet. Afterwards, the suspension was boiled for 10 min and immediately placed in ice for 10 min to prevent DNA degradation and to ensure a higher yield. A second centrifugation (at 10000 rpm for 3 min) was conducted, and 150 μL of supernatant was collected. Finally, the quantity and quality of the extracted DNA were determined using a Nanodrop One spectrophotometer (Thermo Fisher Scientific, USA).

### 2.7 Molecular detection of isolated bacteria

For molecular confirmation, polymerase chain reaction (PCR) and matrix-assisted laser desorption ionization time-of-flight mass spectrometry (MALDI-ToF MS) were performed. PCR was performed using specific primers (Table 2), with the extracted DNA serving as the template. For MALDI-ToF-MS biotyping, the selected bacterial pure cultures (14–18 h old) were sent to the Quality Control Laboratory for Livestock and Livestock Products, Department of Livestock Services, QC Lab Building, Anwar Jang Road, Savar, Dhaka-1343.

**Table 2:** Oligonucleotide sequences, targeted genes, amplicon size, and thermal profile of used primers to detect isolated bacteria from fuchka samples.

Primers	Target gene	Sequence (5′-3′)	Amplicon size (bp)	Thermal Profile						References
				Denaturation		Annealing		Extension		
*Escherichia coli*	*mal*B	F: GACCTCGGTTTAGTTCACAGA	585	35 cycle						[28]
		R: CACACGCTGACGCTGACCA		94 °C	1 m	61 °C	45 s	72 °C	1 m	
*Klebsiella* spp.	*gyr*A	F: CGCGTACTATACATGAACGTA	441	30 cycle						[29]
		R: ACCGTTGATCACTTCGGTCAGG		94 °C	45 s	57 °C	45 s	72 °C	50s	
*K. pneumoniae*	*rpo*B	F: CAACGGTGTGGTTACTGACG	108	35 cycle						[30]
		R: TCTACGAAGTGGCCGTTTTC		94 °C	30s	58 °C	30s	72 °C	30s	
*Staphylococcus* spp.	*16* *s* rRNA	F: GTAGGTGGCAAGCGTTACC	228	30 cycle						[31]
		R: CGCACATCAGCGTCAG		94 °C	30s	55 °C	30s	72 °C	1 m	
*Staphylococcus aureus*	*nuc*	F: GCGATTGATGGTGGATACGGT	279	30 cycle						
		R: AGCCAAGCCTTGACGAACTAAAGC		94 °C	30s	58 °C	30s	72 °C	45 s	
*Enterobacter* spp.	*gro*EL	F: CATACTTCATCAACAAGCCAG	240	32 cycle						This study*
		R: CGAAGCCAGGTGCTTTAAAC		95 °C	30s	58 °C	30s	72 °C	30s	
*Citrobacter* spp.	*asp*C	F: GTTTCGTGCCGATGAACGTC	594	32 cycle						[32]
		R: AAACCCTGGTAAGCGAAGTC		94 °C	30s	58 °C	35	72 °C	45 s	
*Salmonella* spp.	*his*J	F: ACTGGCGTTATCCCTTTCTCTGGTG	496	30 cycle						[33]
		R: ATGTTGTCCTGCCCCTGGTAAGAGA		94 °C	30s	60 °C	30s	72 °C	45 s	

### 2.8 PCR conditions and amplification

All individual PCR reactions for the target genes were performed in a 25 μL reaction volume using Premix Taq (Takara Taq Version 2.0 plus dye, Takara Bio, Japan), gene-specific forward and reverse primers, DNA templates, and nuclease-free water at the optimised concentration. Amplification of all genes was performed at 95 °C for 5 min for initial denaturation and at 72 °C for 7 min for final elongation. Table 2 displays the specific annealing temperatures and cycling parameters for each gene. A 1.5 % agarose gel (Sigma-Aldrich, USA), with Safe Red DNA stain (Hebei SanshiBio-Tech Co. Ltd., China), was utilised for electrophoresis, which was performed at 100 V for 30 min to visualise the PCR product. Finally, the Bio-Rad GelDoc Go Imaging System (Bio-Rad Laboratories Private Limited, Germany) was used to visualise the PCR band.

### 2.9 Antibiotic susceptibility test

Ten commercially available antibiotic discs from nine different classes for Gram-negative isolates and 10 antibiotics from ten different classes for Gram-positive bacterial isolates were used to determine the antibiotic susceptibility profile using the disk diffusion method, following the VET03 guidelines established by the Clinical and Laboratory Standards Institute (CLSI) [34]. Commercially obtained discs (Oxoid Limited, Hampshire, UK) contained the antimicrobials amoxicillin (AMX, 10 μg) (penicillin), azithromycin (AZM, 15 μg) (macrolid), aztreonam (ATM, 30 μg) (monobactum), ceftriaxone (CRO, 30 μg) (cephalosporin), levofloxacin (LEV, 5 μg) (fluroquinolone), gentamicin (CN, 10 μg) (aminoglycoside), nalidixic acid (NA, 30 μg) (fluroquinolone), meropenem (MEM, 10 μg) (carbapenem), cotrimoxazole (SXT, 25 μg) (sulphonamide), and oxytetracycline (OT, 30 μg) (tetracyclin) and were used for Gram-negative bacteria. The discs contained amoxicillin (AMX, 10 μg), tetracycline (TE, 10 μg), chloramphenicol (C, 30 μg) (amphenicol), vancomycin (VA, 30 μg) (glycopeptide), ciprofloxacin (CIP, 5 μg) (fluroquinolone), ceftriaxone (CRO, 30 μg), erythromycin (E, 15 μg) (macrolid), linezolid (LZD, 30 μg) (oxazolidinones), cotrimoxazole (SXT, 25 μg), and gentamicin (CN, 10 μg) to determine the phenotypic antibiotic susceptibility patterns of the Gram-positive bacteria. All bacterial isolates were thawed, streaked onto Tryptone Soy Agar (TSA, HiMedia, India), and incubated at 28 °C for 24 h. Afterwards, the bacteria were inoculated in the nutrient broth, and bacterial suspensions were adjusted to a 0.5 McFarland standard. Then, these suspensions were swabbed onto Mueller-Hinton agar plates (HiMedia, India). Antibiotic discs were carefully placed onto the agar surface, and the plates were inverted and incubated for 24 h at 37 °C. Following incubation, the zone of complete inhibition was determined (in mm). Finally, the diameter of the inhibited zone was compared with the zone-size interpretative table provided by CLSI [34].

### 2.10 Statistical analysis

The data were analysed using SPSS statistical software version 25. The Shapiro-Wilk test was conducted to assess the normality of the data. A one-way ANOVA test was conducted to determine any significant differences in microbial load amongst the categories and individual samples. A threshold *p*-value ≤0.05 was considered statistically significant. Post hoc comparisons were conducted using Tukey's test to pinpoint significant differences between groups. The Kruskal–Wallis test was applied to evaluate significant differences in antibiotic resistance patterns because the corresponding data were not normally distributed.

## 3 Results

### 3.1 Bacterial load assessment

#### 3.1.1 Determination of microbial load by total viable count (TVC)

**Fig. 2:**
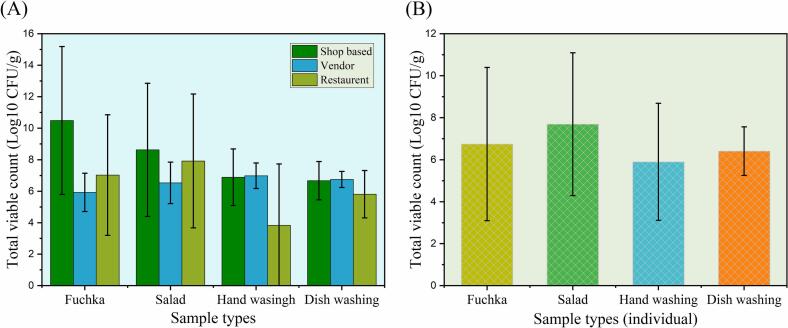
Total Viable Count (TVC) values from fuchka and related materials. (A) fuchka selling point category-wise analysis, (B) individual sample-wise analysis of TVC values of fuchka samples.

The total viable count (TVC) considerably varied across sample types and categories. The highest bacterial load was observed in shop-based fuchka samples (1.5 × 10^15^ CFU/g and 10.5 ± 4.7 mean log CFU/g), whereas the lowest was detected in restaurant hand wash samples [3.30 × 10^4^ CFU/mL (Table S2) and 3.83 ± 3.90 mean log CFU/mL (Table 3)]. Substantial fluctuations in bacterial load were observed in other types of fuchka-related samples (salad, hand wash water, dishwashing water), ranging from 5.81 to 8.63 CFU/g (Fig. 2A). Nevertheless, bacterial load was higher in shop-based fuchka and salad samples. Amongst sample types, the highest microbial load was detected in salad, followed by fuchka, hand wash water, and dishwashing water, with mean CFU/g or CFU/mL of 7.69 ± 3.40, 6.75 ± 3.65, 6.40 ± 1.16, and 5.90 ± 2.78, respectively (Fig. 2B, Table S5). TVC levels did not differ significantly (*p* > 0.05) across shop-based, vendor, and restaurant categories. Even though differences in means were observed, they were not statistically significant (*p* = 0.079) (Table 3).

**Table 3:** Comparison of mean microbial loads (log CFU) across different fuchka selling categories across sample types with Tukey Post Hoc Grouping.

	log (mean ± SD)						
	Category	Fuchka	Salad	Hand wash	Dish wash	Total	*P* value
TVC	Shop-based	10.5 ± 4.7	8.6 ± 4.2	6.9 ± 1.8	6.6 ± 1.2	8.2 ± 3.4^a^	0.079
	Vendor	5.9 ± 1.2	6.5 ± 1.3	6.9 ± 0.8	6.7 ± 0.5	6.5 ± 1.0^a^	
	Restaurant	7.0 ± 3.8	7.9 ± 4.3	3.8 ± 3.9	5.8 ± 1.5	6.1 ± 3.6^a^	
TCC	Shop-based	5.2 ± 2.9	6.5 ± 1.6	4.6 ± 2.9^a^	4.8 ± 2.8	4.9 ± 2.7 ^a^	0.021
	Vendor	1.9 ± 2.2	2.4 ± 4.2	4.9 ± 2.8^a^	4.3 ± 2.5	3.9 ± 2.7 ^ab^	
	Restaurant	0.9 ± 1.9	4.9 ± 7.6	0.0 ± 0.0^b^	2.5 ± 3.4	2.0 ± 3.9 ^b^	
TSC	Shop-based	6.0 ± 1.6	5.3 ± 0.7	4.4 ± 2.6	5.6 ± 1.3	5.3 ± 1.7 ^a^	0.014
	Vendor	2.9 ± 2.8	5.1 ± 0.7	4.2 ± 2.6	3.9 ± 2.3	4.0 ± 2.2 ^ab^	
	Restaurant	4.3 ± 2.6	4.1 ± 4.1	0.9 ± 2.2	2.9 ± 2.6	3.1 ± 3.1 ^b^	

Note: Mean values in the same column followed by different superscripts (ᵃ, ᵇ) indicate significant differences (*p* ≤ 0.05) based on Tukey's HSD post hoc test. Values sharing the same superscript are not significantly different.

#### 3.1.2 Determination of total coliform count (TCC)

**Fig. 3:**
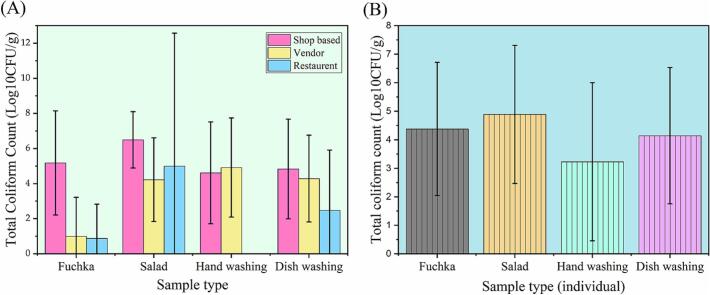
Total Coliform Count (TCC) values from fuchka and related materials. (A) fuchka selling point category-wise analysis, (B) individual sample-wise analysis of TCC values of fuchka samples.

The highest coliform count was detected in shop-based salad (7.75 × 10^8^ CFU/g and 5.03 ± 3.21 mean log CFU/g), whereas the lowest (below countable level) was determined in hand wash water from a restaurant (Table S3). Moreover, TCC values varied across fuchka and other samples, ranging from 0.87 ± 1.95 to 5.17 ± 2.97 log CFU/g (Fig. 3A). Analysis of individual TCC values revealed the highest mean coliform count in salad samples (4.69 ± 4.12 log CFU/g), followed by dishwashing water (3.86 ± 2.92 log CFU/mL), hand wash water (3.18 ± 3.18 log CFU/mL), and fuchka (2.35 ± 3.05 log CFU/g) (Fig. 3B, Table S5). TCC differed significantly (*p* = 0.021) across the selling categories. Tukey's HSD post hoc test revealed that restaurant samples had significantly lower TCC (2.0 ± 3.9ᵇ) than shop-based (4.9 ± 2.7ᵃ) samples (Table 3).

#### 3.1.3 Determination of total staphylococcal count (TSC)

**Fig. 4:**
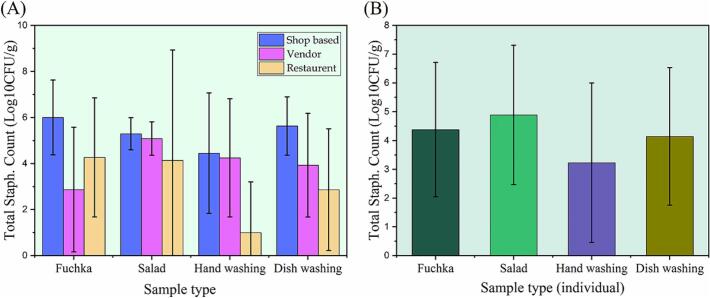
Total Staphylococcal Count (TSC) values from fuchka and related materials. (A) fuchka selling point category-wise analysis, (B) individual sample-wise analysis of TSC values of fuchka samples.

The highest staphylococcal load was detected in fuchka samples from shop-based sources with a mean of 6.00 ± 1.63 log CFU/g (equivalent to 5.5 × 10^8^ CFU/g). In contrast, the lowest was observed in hand wash samples from restaurants (0.99 ± 2.22 mean log CFU/mL) (Table S4 and Table 3). Staphylococcal counts varied across different sample types, including salad, hand wash water, and dishwashing water samples, ranging from 2.86 ± 2.64 log CFU/mL to 5.63 ± 1.27 log CFU/g (Fig. 4A). When comparing individual sample types, salads exhibited the highest average staphylococcal count (4.89 ± 2.42 log CFU/g), followed by fuchka (4.37 ± 2.33 log CFU/g), dishwashing water (4.14 ± 2.30 log CFU/mL), and hand wash water (3.22 ± 2.89 log CFU/mL) (Fig. 4B, Table S5). A significant difference was observed in TSC across groups (*p* = 0.014), indicating that restaurant samples had a significantly lower mean (3.1 ± 3.1ᵇ) than shop-based samples (Table 3).

### 3.2 Bacteriological investigation

The bacteriological investigation of the collected samples revealed that both food samples (crispy fuchka balls with toppings of chickpea, mashed potato, and sour yoghurt) and environmental samples (the seller's hand wash and dishwashing water) were contaminated with various bacterial species. *Escherichia coli*, *Staphylococcus* spp.*, Klebsiella pneumoniae*, *Enterobacter* spp., and *Citrobacter freundii* were the most predominant species, with a group of miscellaneous bacteria in the samples.

#### 3.2.1 Prevalence of different bacteria from fuchka, sellers, and related environmental samples

**Fig. 5:**
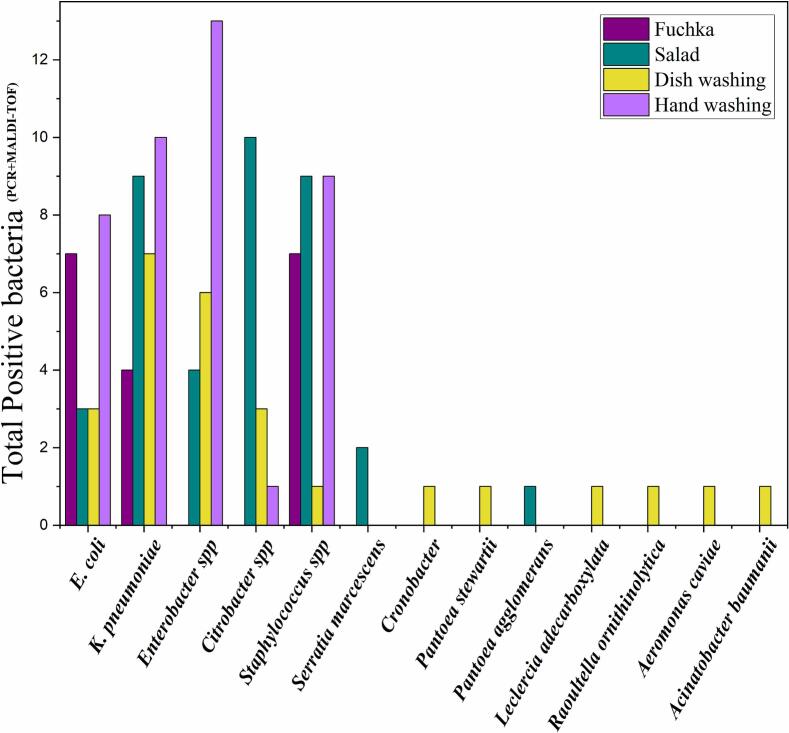
Sample-wise distribution of bacterial isolates (contaminants) from fuchka and fuchka-related materials.

In this study, *Klebsiella pneumoniae*, *Escherichia coli, Enterobacter* spp., *Citrobacter* spp., *Staphylococcus* spp., and some miscellaneous non-targeted bacteria were isolated. Isolates (*n* = 123) encompassed *E. coli* 17 % (*n* = 21), *Klebsiella pneumoniae* 24.4 % (*n* = 30), *Enterobacter* spp. 18.7 % (*n* = 23), *Citrobacter freundii* 11.4 % (*n* = 14), *Staphylococcus* spp. 21.1 % (*n* = 26), *Serratia marcescens* 1.62 % (n = 2), *Cronobacter* sp. 0.8 % (*n* = 1), *Acinetobacter baumanii* 0.8 % (*n* = 1), *Pantoea* spp. 1.62 % (n = 2), *Aeromonas caviae* 0.8 % (*n* = 1), *Raoultella ornithinolytica* 0.8 % (n = 1), and *Leclercia adecarboxylata* 0.8 % (*n* = 1). Fig. 5 and Table 4 present sample-wise and selling category-wise distribution of all isolated bacterial genera.

**Table 4:** Category-wise distribution of isolated bacteria from fuchka and fuchka-related samples.

Isolated bacteria	Total Number	Fuchka selling points					
		Shop		Vender		Restaurant	
		No.	Percentage	No.	Percentage	No.	Percentage
*E. coli*	n = 21	9	43 %	11	52.4 %	1	4.8 %
*K. pneumoniae*	n = 30	16	53.3 %	8	26.7 %	6	20 %
*Enterobacter* spp.	n = 23	9	39 %	8	34.8 %	6	26.1 %
*Citrobacter freundii*	n = 14	4	28.6 %	8	57.1 %	2	14.3 %
*Staphylococcus* spp.	n = 26	16	61.5 %	7	27 %	3	11.5 %
*Serratia marcescens*	n = 2	2	–	–	–	–	–
*Cronobacter* spp.	n = 1	1	–	–	–	–	–
*Pantoea stewartii*	n = 1	–	–	1	–	–	–
*Pantoea agglomerans*	n = 1	–	–	1	–	–	–
*Leclercia adecarboxylata*	n = 1	–	–	1	–	–	–
*Raoultella ornithinolytica*	n = 1	–	–	2	–	–	–
*Aeromonas caviae*	n = 1	–	–	–	–	1	–
*Acinatobacter baumanii*	n = 1	–	–	–	–	1	–

#### 3.2.2 Cultural, gram staining, and biochemical characteristics of different isolated bacteria from fuchka samples

Cultural morphology, biochemical characterisation, and Gram staining were used to identify the isolated bacteria. The cultural characteristics of each bacterial type in fuchka and related samples were examined to determine size, shape, and colony morphology on various bacteriological media. Pure cultures were obtained from mixed cultures utilising the repeated streak plate method with different simple and selective solid media. Table S6 and Fig. S1 provide the cultural characteristics of *E. coli*, *Klebsiella pneumoniae*, *Staphylococcus* spp., *Enterobacter* spp., and *Citrobacter* spp. grown on the media. The methyl red test yielded positive results for *E. coli* and *Staphylococcus* spp., while *Klebsiella pneumoniae* was negative. All isolates were tested positive for the catalase test, with gas bubble formation. Table S7 provides additional details about the biochemical properties, including the sugar fermentation test and other relevant information.

#### 3.2.3 Molecular detection of isolated bacteria from fuchka samples

**Fig. 6:**
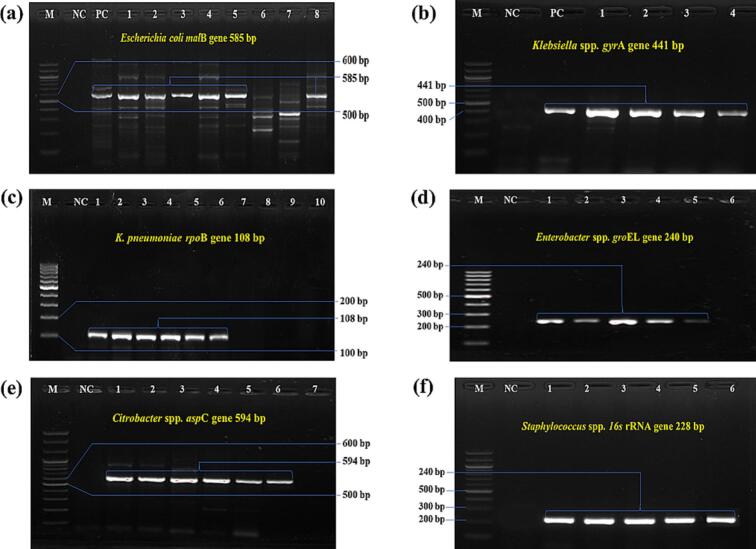
PCR amplification of genus- and species-specific primers used to detect isolated bacteria. a) PCR detection of *E. coli*, targeting *mal*B gene with amplicon size 585 bp; b) genus-specific PCR detection of *Klebsiella* spp., using gyrA gene and amplicon size 441 bp; c) *K. pneumoniae*, targeting 108 bp sequence of *rpo*B gene; d) *gro*EL gene-based PCR detection of *Enterobacter* spp., with amplicon size of 240 bp; e) PCR detection of *Citrobacter* spp., targeting *asp*C gene with 594 bp; f) PCR detection of *Staphylococcus* spp., using *16* *s* rRNA gene with 228 bp amplicon. In all cases, M: 100 bp DNA ladder; NC: negative control; PC: positive control).

One hundred twenty-three bacterial isolates were confirmed using PCR and MALDI-TOF-MS assay. *E. coli*, *Klebsiella pneumoniae, Enterobacter* spp., *Citrobacter* spp., and *Staphylococcus* spp. were detected by PCR (Fig. 6). Suspected culture-positive *Salmonella* spp. were negative both in PCR and MALDI-TOF, which were later confirmed as *Citrobacter freundii* by PCR and MALDI-TOF. None of the *Staphylococcus* spp. was positive for the *nuc* gene*.*

#### 3.2.4 Results of MALDI-ToF-MS analysis

A score over 2.3 indicates ‘highly probable species identification’, while a score between 2 and 2.299 reveals ‘secure genus identification with probable species identification’. Moreover, a score between 1.7 and 1.999 demonstrates ‘probable genus identification’, and a score below 1.7 depicts ‘unreliable identification’. Various species of isolated bacteria, genera, and some non-specific bacteria were identified and confirmed by MALDI-TOF. Table S8 presents the detailed results of the MALDI-TOF MS.

### 3.3 Antibiogram profiling

#### 3.3.1 Antibiotic susceptibility result of isolated bacteria

**Fig. 7:**
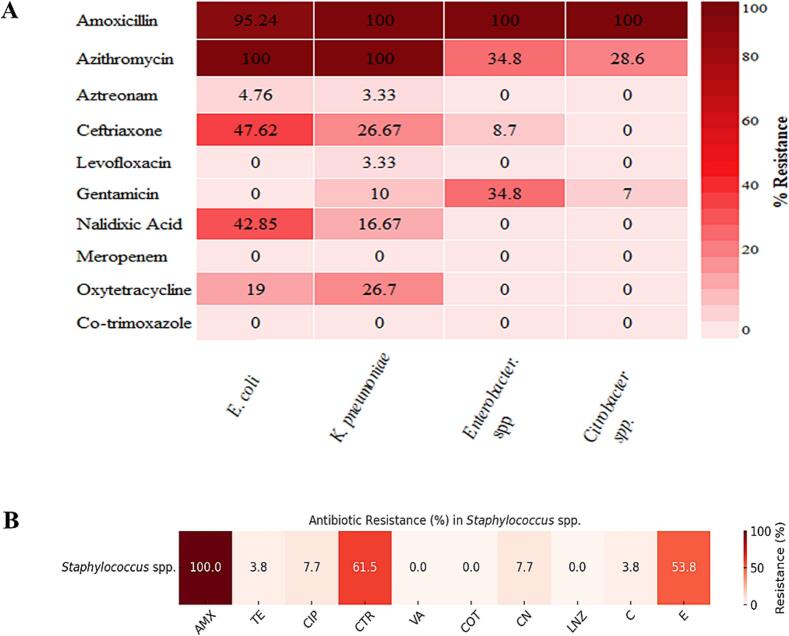
Heatmap showing antimicrobial resistance percentages of all bacterial isolates. (A) Gram-negative bacterial isolates (*E. coli*, *K. pneumoniae*, *Enterobacter* spp., and *Citrobacter* spp.); and (B) Gram-positive bacterial isolate (*Staphylococcus* spp.).

Of the Gram-negative bacterial isolates, 95.24 % of *E. coli*, 100 % of *K. pneumoniae* and *Enterobacter* spp., and *Citrobacter freundii* were resistant to amoxicillin. Both *E. coli* and *K. pneumoniae* isolates demonstrated 100 % resistance to azithromycin, whereas 34.8 % (8 isolates) of *Enterobacter* spp. were resistant, and 60 % (14 isolates) exhibited intermediate sensitivity. Within the aminoglycoside group, 100 % of *E. coli,* 76.67 % of *K. pneumoniae*, and 35 % of *Citrobacter freundii* isolates were found to be sensitive to gentamicin. However, none of the *Enterobacter* spp. isolates illustrated complete sensitivity, with all bacterial isolates being 100 % sensitive to co-trimoxazole (Fig. 7A). For the Gram-positive isolates (*Staphylococcus* spp.), amongst 26 *Staphylococcus* spp. isolates, 100 % were resistant to amoxicillin, with all being sensitive to co-trimoxazole and linezolid. Fig. 7B depicts that for tetracycline and ciprofloxacin, 7.7 % (2 out of 26 isolates) were resistant, and one isolate exhibited intermediate sensitivity, indicating bacterial isolates' exposure to commonly used antibiotics, either through food animals, human waste, or environmental contamination. Tables S9 and S10 provide the detailed antibiogram profile for both Gram-negative and Gram-positive bacteria. A statistical comparison of resistance profiles using the Kruskal-Wallis test did not reveal any significant differences amongst bacterial genera for most antibiotics (*p* > 0.05), suggesting a uniformly high resistance pattern across the tested isolates.

#### 3.3.2 Analysis of multidrug resistance (MDR) patterns and multiple antibiotic resistance (MAR) index of isolated bacteria

Multidrug resistance (MDR) was detected in 57 % of *E. coli*, 90 % of *K. pneumoniae*, 8.7 % of *Enterobacter* spp., and 50 % of *Staphylococcus* spp. isolates, demonstrating noteworthy antibiotic resistance, particularly amongst Gram-negative pathogens. However, no *Citrobacter freundii* isolate was identified as MDR. The multiple-antibiotic resistance (MAR) index analysis posited that the MAR index values ranged from 0.2 to 0.6 in both *E. coli* and *K. pneumoniae*. Nevertheless, they ranged from 0.1 to 0.3, 0.1 to 0.2, and 0.1 to 0.4 for *Enterobacter* spp., *Citrobacter* spp., and *Staphylococcus* spp., respectively. Thus, all the *E. coli* and *K. pneumoniae* isolates exhibited the MAR index value of ≥0.2. Nonetheless, 60.87 %, 28.57 %, and 80.77 % of isolates of *Enterobacter* spp., *Citrobacter* spp., and *Staphylococcus* spp. had the value of ≥0.2, respectively. Only one *Enterobacter* spp. isolate exhibited a MAR index of 0.3, whereas no *Citrobacter* spp. isolates surpassed a MAR value of 0.2. The highest MAR index (0.6) was detected in a single *K. pneumoniae* isolate (Table 5). Table 6 summarises key antibiotics with the highest resistance rates with MDR implications across bacterial isolates.

**Table 5:** Multiple antibiotic resistance (MAR) index analysis of isolated bacteria from fuchka samples.

Bacterial genera	Antibiotic resistance combinations	MAR Index	No. of resistance classes	Strand point
*E. coli* (n = 21)	AMX, AZM	0.2	2	Drug resistant
	AZM, CRO, OT	0.3	3	MDR
	AMX, AZM, CTR	0.3	3	MDR
	AMX, AZM, T	0.3	3	MDR
	AMX, AZM, OT, NA	0.4	4	MDR
	AMX, AZM, CTR, NA	0.4	4	MDR
	AMX, AZM, CTR, OT	0.4	4	MDR
	AMX, AZM, ATM, CTR, NA	0.5	5	MDR
*Klebsiella pneumoniae* (n = 30)	AMX, AZM	0.2	2	Drug resistant
	AMX, AZM, CTR	0.3	3	MDR
	AMX, AZM, CN	0.3	3	MDR
	AMX, AZM, NA	0.3	3	MDR
	AMX, AZM, OT	0.3	3	MDR
	AMX, AZM, LEV	0.3	3	MDR
	AMX, AZM, CTR, NA	0.4	4	MDR
	AMX, AZM, CTR, CN	0.4	4	MDR
	AMX, AZM, NA, OT	0.4	4	MDR
	AMX, AZM, CTR, OT	0.4	4	MDR
	AMX, AZM, ATM, CTR, NA, OT	0.6	6	MDR
*Enterobacter* spp. (n = 23)	AMX	0.1	1	Drug resistant
	AMX, AZM	0.2	2	Drug resistant
	AMX, CN	0.2	2	Drug resistant
	AMX, CTR	0.2	2	Drug resistant
	AMX, AZM, CN	0.3	3	MDR
*Citrobacter freundii* (n = 14)	AMX	0.1	1	Drug resistant
	AMX, AZM	0.2	2	Drug resistant
	AMX, CN	0.2	2	Drug resistant
*Staphylococcus* spp. (n = 26)	AMX	0.1	1	Drug resistant
	AMX, CTR	0.2	2	Drug resistant
	AMX, CN	0.2	2	Drug resistant
	AMX, E	0.2	2	Drug resistant
	AMX, CIP	0.2	2	Drug resistant
	AMX, CTR, E	0.3	3	MDR
	AMX, CN, E	0.3	3	MDR
	AMX, CTR, CN, E	0.4	4	MDR
	AMX, TE, CTR, E	0.4	4	MDR
	AMX, CTR, C, E	0.4	4	MDR
	AMX, CIP, CTR, C	0.4	4	MDR

Note: AMX = Amoxicillin, AZM = Azithromycin, ATM = Aztreonam, TE = Tetracycline, CIP = Ciprofloxacin, CTR = Ceftriaxone, VA = Vancomycin, COT = Cotrimoxazole, CN = Gentamicin, LEV = Levofloxacin, OT = Oxytetracycline, NA = Nalidixic acid, C = Chloramphenicol, E = Erythromycin, MDR = Multi durg resistance.

**Table 6:** Summary of key antibiotics with highest resistance rates and MDR implications across bacterial isolates.

Antibiotic	Classes	Bacterial genera showing resistance	Associated MDR cases	Highest MAR index observed	Public health concern
Amoxicillin (AMX)	Beta-lactam	*E. coli*, *K. pneumoniae*, *Enterobacter* spp., *Citrobacter freundii*, *Staphylococcus* spp.	Yes (multiple genera)	0.6	Widespread resistance; base antibiotic in treatment
Azithromycin (AZM)	Macrolide	*E. coli*, *K. pneumoniae*, *Citrobacter freundii*	Yes	0.6	Frequently used for respiratory and GI infections
Ceftriaxone (CTR)	Cephalosporin (3rd gen)	*E. coli*, *K. pneumoniae*, *Staphylococcus* spp.	Yes	0.6	Last-resort for many systemic infections
Oxytetracycline (OT)	Tetracycline	*E. coli*, *K. pneumoniae*	Yes	0.6	Common in veterinary and human use
Nalidixic Acid (NA)	Quinolone	*E. coli*, *K. pneumoniae*	Yes	0.6	First-gen quinolone; resistance indicates pressure
Gentamicin (CN)	Aminoglycoside	*K. pneumoniae*, *Enterobacter* spp., *Staphylococcus* spp.	Yes	0.4	Hospital use; rising resistance is alarming
Erythromycin (E)	Macrolide	*Staphylococcus* spp.	Yes	0.4	Frequently used; resistance reduces oral options
Chloramphenicol (C)	Amphenicol	*Staphylococcus* spp.	Yes	0.4	Restricted use; MDR presence is concerning
Aztreonam (ATM)	Monobactam	*E. coli*, *K. pneumoniae*	Yes	0.6	Used against Gram-negative bacteria

## 4 Discussion

Fuchka, a popular South Asian street food, is widely enjoyed across Bangladesh and neighbouring regions by people of all socioeconomic backgrounds [6]. Fuchka comprises a hollow, crispy shell made from fried wheat flour, filled with spicy chickpeas and mashed potatoes. It is typically garnished with yoghurt, thin slices of onion and cucumber, shredded boiled eggs, and served with tangy tamarind water [2]. Fuchka may have originated in the state of Bihar, India, with the name ‘fuchka’ being utilised in Bangladesh, Nepal, Jharkhand, and West Bengal [24]. However, the food safety issues, particularly microbial foodborne illnesses, antimicrobial resistance, and MDR bacteria, have received considerable attention as a major public health concern regarding street foods, such as fuchka [13,14].

**Fig. 8:**
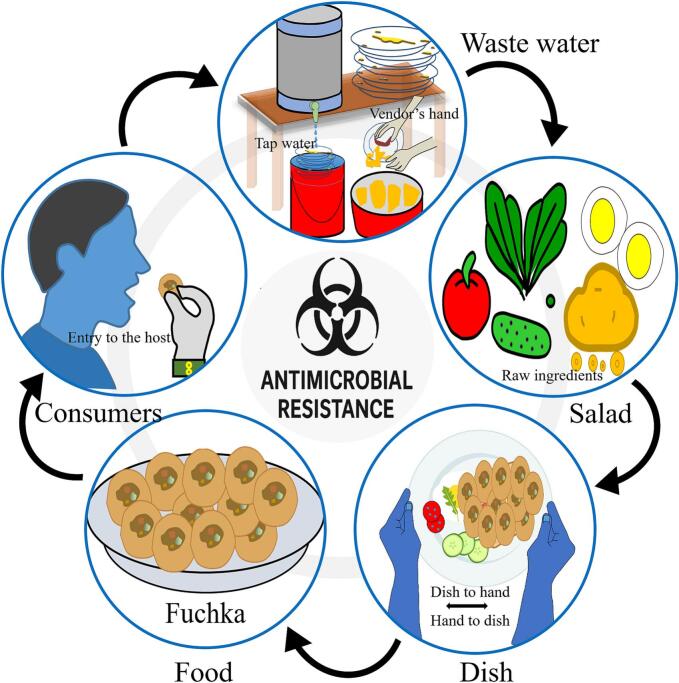
The potential transmission pathway of antimicrobial resistant bacteria from environmental sources (i.e., contaminated water, vegetables) to fuchka, fuchka-handlers, and ultimately to consumers.

Street vendor food symbolises an informal food system with inadequate regulatory supervision. To this end, the One Health framework has been highly relevant, because inadequate hygiene, unsafe water utilisation, and inadequate waste disposal can foster the multidirectional transmission of antimicrobial-resistant (AMR) bacteria amongst humans, animals, and the environment [35]. This process, as observed in current study's context is illustrated in Fig. 8, which depicts the potential transmission pathway of AMR from environmental sources (such as contaminated water) to food, food handlers, and ultimately to consumers. Additionally, in many low- and middle-income countries (LMICs), including Bangladesh, food safety has been under-prioritised. Even though foodborne AMR has increasingly received global attention, implementing safeguards in LMICs is often limited to theoretical frameworks instead of operational policies [36].

The microbiological safety of street food, particularly fuchka, poses a pivotal public health concern. Accordingly, the present study evaluated the microbiological safety of fuchka by determining the total viable count (TVC), total coliform count (TCC), and total staphylococcal count (TSC). It also focused on the isolation, molecular confirmation, and antibiogram of common foodborne bacteria. Sixty samples, including fuchka, salad, hand wash water, and dishwashing water were collected from three types of selling facilities (5 sites per category) within Mymensingh City Corporation.

Of the four sample types tested (fuchka, salad, hand wash water, and dishwashing water), shop-based fuchka samples had the highest mean TVC (10.49 ± 4.69 CFU/g), whereas the lowest count was detected in hand wash water samples from a restaurant (3.83 ± 3.90 CFU/mL). Sample-wise TVC analysis resulted in the following mean count values: 7.69 ± 3.40 (salad), 6.75 ± 3.65 (fuchka), 6.40 ± 1.16 (hand wash water), and 5.90 ± 2.78 (water used in dishwashing) CFU/g or CFU/mL. Even though Hasan et al. [2] detected viable bacteria in all types of samples, in the present study, a few hand wash water samples from restaurants had either no viable bacteria or extremely low counts, likely due to improved sanitary practices. Moreover, Hasan et al. [2] reported the highest TVC in hand wash water samples (6.18 ± 1.70 log CFU/g). Conversely, the present study exhibited the lowest count of 5.90 ± 2.78 mean CFU/mL, which may be associated with the inclusion of different categories of fuchka facilities. Interestingly, the mean CFU/g of hand wash water samples from the vendor in the present study was 6.98 ± 0.81, aligning with the findings of Hasan et al. [2]. These results are similar to those of previous studies, including one from Tangail district, where TVC values ranged from 2.5 × 10^6^ to 8.9 × 10^8^ CFU/g. Yadav and Yadav [37] reported similar TVC of masala pani and solid matter masala values in Nepal, ranging from 90 to 182 × 10^5^ to 50–121 × 10^5^ and from 80 to 130 × 10^1^ to 46–118 × 10^5^ from vendors of congested and non-congested locations, respectively, while analysing 120 panipuri samples.

TCC analysis exhibited the highest values in shop-based salad samples (5.03 ± 3.21 CFU/g), and the lowest values (nil) in hand wash water samples from a restaurant. Sample-wise TCC values were highest in salad samples (4.69 ± 4.12 CFU/g), followed by dishwashing water (3.86 ± 2.92 CFU/mL), hand wash water (3.18 ± 3.18 CFU/mL), and fuchka (2.35 ± 3.05 CFU/g). These results align with earlier work by Hasan et al. [2] who detected coliform in 67.5 % (mean of 4.10 ± 0.73 log CFU/g) of the total samples, where 40 % were from fuchka (4.56 ± 1.68 log CFU/g), 20 % from salad (3.70 ± 1.38 log CFU/g), and 40 % from shredded eggs (3.21 ± 1.00 CFU/g).

TSC enumeration demonstrated that the highest values were detected in shop-based fuchka samples (6.00 ± 1.63 CFU/g), while the lowest values were found in hand-wash water from restaurants (0.99 ± 2.22 CFU/mL). Sample-wise TSC values were highest in salad samples (4.89 ± 2.42 CFU/g), followed by fuchka (4.38 ± 2.33 CFU/g), dishwashing water (4.14 ± 2.39 CFU/mL), and hand-wash water (3.22 ± 2.89 CFU/mL). Hasan et al. [2] detected *Staphylococcus* spp. contamination (average 5.15 ± 1.22 log CFU/g) in all hand-wash samples (100 %), with the lowest contamination in shredded eggs (70 %; mean 3.82 ± 1.50 log CFU/g). Afrin et al. [22] presented the TSC values of various street food items, ranging from 5.0 × 10^5^ - 4.6 × 10^6^ CFU/mL to 3.1 × 10^4^–6.6 × 10^5^ CFU/mL in street-vended foods and restaurant foods, respectively.

Statistically significant differences existed in total coliform count (TCC, *p* = 0.021) and total staphylococcal count (TSC, *p* = 0.014) across fuchka selling categories. Restaurant samples exhibited the lowest TCC (2.0 ± 3.9ᵇ), significantly lower than shop-based samples (4.9 ± 2.7ᵃ), with vendor samples (3.9 ± 2.7ᵃᵇ) being intermediate. Likewise, shop-based samples exhibited the highest TSC (5.3 ± 1.7ᵃ), significantly surpassing restaurant samples (3.1 ± 3.1ᵇ), with vendors falling in between (4.0 ± 2.2ᵃᵇ). Even though total viable count (TVC) did not differ significantly (*p* = 0.079), the highest mean TVC was observed in shop-based samples (8.2 ± 3.4), indicating an elevated microbial risk. These variations in microbial loads convincingly depict enormous fluctuations in hygiene and sanitation practices across selling environments. A higher TCC in shop-based samples suggests higher faecal contamination, likely attributed to poor hand hygiene, unsafe water, or cross-contamination during food preparation. However, elevated TSC levels indicate inadequate control of staphylococcal contamination, possibly due to improper handling or storage. Even though vendor samples demonstrated moderate contamination levels, likely due to inconsistent hygiene measures, restaurant samples consistently exhibited lower microbial loads, suggesting more regulated and safer food-handling practices. According to ICMSF standards [27], only 13.33 % of fuchka and dishwashing water samples, and 6.67 % of salad and hand wash water samples, complied with acceptable microbiological thresholds; the remainder exceeded these limits, demonstrating widespread contamination and substandard hygiene. All fuchka samples from shop-based and vendor sources exceeded the acceptable TCC levels, except for those from vendor sources. Conversely, restaurant samples typically met the standards, except for salad. Although sample-wise mean TSC values were within acceptable limits (<10^5^ CFU), all category-wise shop-based samples and vendor-type salad exceeded this threshold except for hand-wash samples. Only restaurant samples were within acceptable or even satisfactory levels (<10^3^ CFU) [27], indicating that fuchka from restaurant-type facilities exhibited comparatively lower microbiological risk than those from shops or street vendors.

Amongst all category samples, the most abundant and frequently isolated bacteria were *Klebsiella pneumoniae* (*n* = 30), followed by *Staphylococcus* spp. (*n* = 26), *Escherichia coli* (*n* = 21), *Citrobacter* spp. (*n* = 14), and *Enterobacter* spp. (*n* = 23). The less frequently isolated bacterial genus and/or species comprised *Cronobacter* spp.*, Pantoea stewartii, Leclercia adecarboxelata, Roultella ornithinolytica, Aeromonas caviae*, *Acinetobacter baumanii,* and *Pantoea agglomerans*. Cultural, biochemical, and morphological characterisation was conducted using the description reported by Patra et al. [26]. Reports covering only fuchka-based bacterial detection are rare in Bangladesh. Nonetheless, Hasan et al. [2] reported the presence of *E. coli* and *Staphylococcus* spp. in fuchka samples sold by various vendors in the Mymensingh city corporation area. Moreover, Gaia et al. [38] found *Listeria monocytogenes, Clostridium botulinum, E. coli, Salmonella* spp., *Yersinia enterocolitica, Staphylococcus aureus, Shigella* spp., *Bacillus cereus,* and *Campylobacter jejuni* in street foods. Similarly, Islam et al. [39] detected bacterial pathogens, such as *Pseudomonas nitroreducens*, *Citrobacter braakii*, and *Klebsiella pneumoniae* subsp. *pneumoniae*, and *Serratia marcescens*, at the molecular level in street foods. Rahman et al. and Jahan et al. [3,4] detected *Salmonella* spp. and *E. coli* (O157, O111, O26), coliform bacteria, *Enterococcus* spp., *Listeria* spp., *Yersinia* spp., *Enterobacter sakazakii,* and *Staphylococcu*s spp. in street foods in Dhaka city. Furthermore, Khalif et al. [5] and Aktar et al. [6] found *Klebsiella* spp. Environmental contamination plays a pivotal role in the contamination of street food. Pathogenic contamination sources include cross-contamination of used water through the mixing of waste with water or the use of water from unacceptable sources for cleaning utensils and washing raw materials [40]. The detection of bacteria, such as *Aeromonas caviae* and *Cronobacter*, in dishwashing water highlights the potential use of river water or contaminated water to wash utensils, because samples were collected from vendors in a riverside park, where several researchers previously reported the presence of *Aeromonas caviae* in river water [41]. Additionally, the high prevalence of *E. coli, K. pneumoniae, and Enterobacter* spp. may be due to unhygienic food handling [39]. The presence of *Staphylococcus* spp. both in fuchka and hand wash water may be associated with contaminated yoghurt and improper hand hygiene [42].

The molecular detection using PCR relied on established protocols from previous studies. *E. coli* was confirmed by a 585 bp amplicon of the *malB* gene [28], whereas *Staphylococcus* spp. was validated by a 228 bp fragment of the 16S rRNA gene [31]. *Enterobacter* spp. and *Citrobacter* spp. were identified by 240 bp (*groEL*) and 594 bp (*aspC*) amplicons, respectively [32]. Even though *Staphylococcus aureus* detection was explored by utilising the *nuc* gene [31], none of the *Staphylococcus* spp. were positive. Presumptive *Salmonella* spp. colonies (black on SS agar) were tested by amplifying a 496 bp product of the *hisJ* gene [33].

The most common genera were confirmed by PCR-based molecular identification. Additionally, the MALDI-ToF-MS analysis was also conducted to resolve inconsistencies in PCR results (such as non-specific bands or missing amplification). Thus, the additional genera were confirmed as *Cronobacter* spp.*, Pantoea stewartii, Leclercia adecarboxylata, Roultella ornithinolytica, Aeromonas caviae*, *Acinetobacter baumanii,* and *Pantoea agglomerans*. Most isolates yielded a score value ≥2, which exhibited ‘secure genus identification’ [43], except for isolate 2(1): *Pantoea stewartii* (score value 1.84), isolate 4(1): *Cronobacter* spp. (score value 1.77), isolate 7(1): *Enterobacter cloacae* (score value 1.75), isolate 8(12): *Staphylococcus warneri* (score value 1.98), and isolate 9(9): *Pantoea agglomerans* (score value 1.73). The score exceeding 1.7 and less than 1.99 demonstrates ‘probable genus identification’ [43].

Over the past two decades, Bangladesh has experienced a growing burden of AMR, which is primarily associated with the indiscriminate and widespread use of antibiotics in agriculture and livestock production [44]. The non-therapeutic and prophylactic use of antimicrobials in livestock fosters the emergence of resistance, as it enters into the human food chain through contaminated meat, eggs, milk, milk products, or wastewater [19]. Amongst the isolated bacteria, *E. coli*, *K. pneumoniae*, *Enterobacter* spp., *Citrobacter* spp., and *Staphylococcus* spp. were subjected to phenotypic antibiotic resistance/susceptibility tests using the disc diffusion method, which comprised various antimicrobial classes, including aminoglycosides, sulfonamides, monobactams, and beta-lactams. An antibiogram posited that all *E. coli* isolates (95.25 %) exhibited resistance against amoxicillin and (100 %) against azithromycin but were sensitive to gentamicin and cotrimoxazole. However, all *K. pneumoniae* isolates (100 %) developed resistance to amoxicillin and azithromycin but were sensitive to cotrimoxazole. Isolates of *Citrobacter freundii* and *Enterobacter* spp. demonstrated high sensitivity (100 %) towards meropenem, oxytetracycline, and cotrimoxazole, whereas complete resistance to intermediate sensitivity was found towards amoxicillin (100 %) and gentamicin. All *Staphylococcus* spp. isolates exhibited 100 % sensitivity towards vancomycin, cotrimoxazole, and linezolid, with resistance to amoxicillin. Likewise, Sultana et al. [14] reported that *E. coli* was resistant to streptomycin (85.71 %), ceftriaxone (100 %), erythromycin (100 %), meropenem (100 %), and gentamicin (71.42 %) while analysing 42 street food samples of 6 categories collected from 7 different areas of Dhaka city. Furthermore, Islam et al. [23] reported considerably low resistance of *Salmonella* spp., *Klebsiella* spp., *Vibrio* spp., and *Staphylococcus aureus* towards imipenem, meropenem, and amikacin. Nonetheless, a higher resistance against azithromycin and ciprofloxacin, and for *S. aureus*, 67 % of isolates demonstrated methicillin resistance.

Hasan et al. reported that 41.66 % of *E. coli* were resistant to tetracycline, which was confirmed by the consistent amplicon size of 577 bp of the tetracycline-resistant *tet*A gene [2]. *E. coli* and *Klebsiella* spp. were isolated in another study, where street foods from the Dinajpur district of Bangladesh were investigated. The study's author reported sensitivity to ciprofloxacin and resistance to cefixime, cefalexin, erythromycin, fusidic acid, cefuroxime, and aztreonam. For *Staphylococcus* spp., sensitivity was observed towards ciprofloxacin, gentamicin, and neomycin, and the resistance was noted to erythromycin and fusidic acid [2]. Moreover, Aktar et al. [6] demonstrated that streptomycin, gentamicin, tetracycline, and neomycin were sensitive for both the isolated *E. coli* and *Klebsiella* spp. Similarly, vancomycin, penicillin, erythromycin, amoxicillin, and ampicillin were found to be resistant to *E. coli* isolates. In contrast, vancomycin, azithromycin, penicillin, erythromycin, amoxicillin, and ampicillin were detected as being resistant to *Klebsiella* spp. [6]. In Malaysia, Zulfakar et al. [45] isolated 100 % amoxicillin, ceftriaxone and trimethoprim-sulfamethoxazole-sensitive *Citrobacter freundii* from RTE beverage samples.

Multidrug resistance (MDR) pattern analysis revealed that all *E. coli* isolates (57.1 %) were found to be MDR. Nonetheless, 18 out of 30 (60 %) isolates of *K. pneumoniae,* 1 out of 23 *Enterobacter* spp., and 12 out of 26 (46.1 %) *Staphylococcus* spp. isolates were detected as MDR. Likewise, Samy et al. [44] reported that most *E. coli* isolates exhibited multidrug resistance, and resistance genes (*bla*TEM and *tet*A) were detected in all the tested amoxicillin and Oxytetracycline-resistant *E. coli* isolates from various food samples. Adhikari et al. [46] detected MDR *E. coli* while analysing the approximate scenario of AMR in *E. coli* and *Salmonella* species from street-vended RTE chutney samples (*n* = 150) in Bharatpur, Nepal. Regarding MDR *Klebsiella* spp., Hartantyo et al. [47] reported MDR *K. pneumoniae* isolates from RTE poultry-origin foods, fresh raw vegetables, and the livers of poultry species and pigs. Therefore, fresh and raw poultry products could harbour MDR *K. pneumoniae*, as mentioned by Silva-Bea et al. [48].

The multiple antibiotic resistance (MAR) index values illustrated that 12 isolates of *E. coli* (*n* = 21) scored ≥0.2, with the highest MAR value of 0.5 (1/21). Moreover, all *K. pneumoniae* isolates (*n* = 30) varied from 0.2 (12/30) to 0.6 (1/30); however, the highest MAR index was observed in *Staphylococcus* spp. (*n* = 26) isolates as 0.4, which was observed in 5 isolates. High-risk sources of food contamination can be identified via the MAR indexing of bacteria, including *E. coli* and *Klebsiella* spp. [49]. Mir et al. [20] reported MAR index values of *E. coli* ranging from 0.45 to 0.81 when analysing 150 chicken meat samples microbiologically in Zahedan, Southeastern Iran. The ≥0.2 MAR index values demonstrated that the sampling areas were heavily contaminated with resistant bacteria, which could pose a serious public health concern [49]. Urmi et al. [42] declared that 75 % of *Staphylococcus* spp. isolates had MAR values ranging from 0.2 to 0.6 in RTE fast food. Amoxicillin exhibited resistance across all five genera studied, underscoring its reduced efficacy due to overuse and misuse. Azithromycin and ceftriaxone were commonly detected in MDR profiles, underscoring the emergence of resistance to critically important antibiotics. The MAR index reached a high of 0.6 in some isolates, particularly in *E. coli* and *K. pneumoniae*, indicating sustained antimicrobial exposure and high-risk contamination sources. Additionally, resistance to hospital-grade antibiotics, such as gentamicin and aztreonam, poses a serious clinical concern, particularly in low-resource settings [15].

The present study accentuated the pivotal need for implementing localised food safety regulations targeting street food vendors. Table S11 depicts regional differences in contamination, indicating the need for a coordinated street food surveillance program in Bangladesh. Key interventions must include mandatory training programs for food handlers that address hygienic practices, highlight formal licensing systems, emphasize routine inspection of food vending sites, and establish infrastructure, such as access to clean water and proper waste disposal at designated vending zones. Furthermore, incorporating antimicrobial resistance (AMR) surveillance into existing public health frameworks could foster early detection and control of resistant foodborne pathogens, thereby reinforcing food safety governance and supporting One Health-informed public health policymaking [18]. Moreover, by contextualising street food safety as a One Health challenge, these interventions can substantially reduce risks to human health and environmental and animal health systems involved in food production.

Nevertheless, the present study has several limitations. The sample size, though adequate for preliminary analysis, may fail to capture regional variability across Bangladesh. Moreover, the sampling was restricted to the Mymensingh City Corporation area, which limited the geographic generalisability of the findings. Additionally, temporal factors, such as seasonal variation and time of day, were not considered, even though they may have impacted microbial contamination levels. Finally, the scope of the study was limited to bacterial assessment. It did not include viral or parasitic pathogens, which are also closely associated with comprehensive evaluations of street food safety.

## 5 Conclusion

The higher microbial loads detected in shop-based fuchka samples may result from moderate foot traffic combined with inadequate sanitation, fostering cross-contamination. Conversely, vendor-based stalls, despite having inferior infrastructure, may benefit from shorter storage times and fewer customer interactions, whereas restaurants typically maintain improved hygiene standards and cleaning protocols. Pathogens, including *Klebsiella pneumoniae*, *Staphylococcus* spp., *Escherichia coli*, *Citrobacter* spp., and *Enterobacter* spp.*,* were frequently isolated, with most exhibiting resistance to amoxicillin. Notably, *K. pneumoniae* exhibited resistance to both amoxicillin and azithromycin, raising serious public health concerns. The comparatively higher rates of multidrug resistance, particularly amongst *E. coli* and *K. pneumoniae*, depict the growing threat of antimicrobial resistance (AMR) in everyday food sources. Furthermore, the MAR index demonstrates the indiscriminate use of antibiotics and emphasises contaminated food as a potential vehicle for resistant bacteria. These findings underscore the urgent need for integrated interventions to safeguard food safety and public health. A One Health approach, fostering collaboration across human, animal, and environmental sectors, is pivotal. The critical steps must include improving regulatory oversight, elevating hygiene in street food preparation, and facilitating responsible antibiotic use in both medical and agricultural settings. Additionally, the regulatory agencies must establish a coordinated multisectoral surveillance system to monitor microbial contamination and AMR trends across environments.

## CRediT authorship contribution statement

**Bushra Benta Rahman Prapti:** Writing – original draft, Visualization, Software, Methodology, Investigation, Formal analysis, Data curation. **Md. Tanjir Ahmmed:** Investigation, Formal analysis, Data curation. **Nishita Ghosh Proma:** Methodology, Investigation. **Durratul Zanan Aunu:** Methodology, Investigation. **Shumia Islam Shampa:** Methodology, Investigation. **Aminur Rahman:** Methodology, Investigation. **Md. Shafiqul Islam:** Writing – review & editing, Validation, Investigation. **Mahbubul Pratik Siddique:** Writing – review & editing, Validation, Supervision, Resources, Project administration, Funding acquisition, Conceptualization.

## Declaration of competing interest

There is no conflict of interest as declared by all the authors.

## Data Availability

Data will be made available on request.

## Appendix A Supplementary data

Supplementary material 1 Image 1

Supplementary material 2 Image 2
