# Supplementary material for: Bacterial load assessment and multi-drug resistant Bacteria isolation from Fuchka in Mymensingh City, Bangladesh
Source: One Health. 2025 Aug 18;21:101170. doi: 10.1016/j.onehlt.2025.101170 (PMC12396293; doi:10.1016/j.onehlt.2025.101170)
Supplement: Supplementary file 1 — Supplementary material 1 [file mmc1.docx]

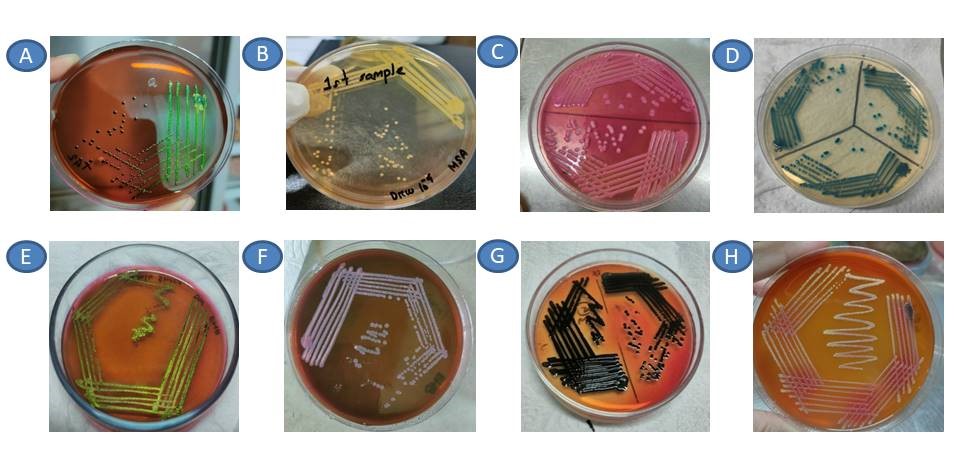


**Figure S1:** Growth of suspected bacteria on different agar media A) *E. coli* on EMB agar, B) *Staphylococcus* spp. on MSA agar, C) *Klebsiella pneumoniae* on MacConkey agar, D) *Klebsiella pneumoniae* on HiCrome UTI agar, E) *Enterobacter* spp. on EMB agar, F) *Enterobacter* spp. on EMB agar. G) *Citrobacter* spp. on SS agar, H) *Citrobacter* spp. on XLD agar
