# Supplementary material for: Bacterial load assessment and multi-drug resistant Bacteria isolation from Fuchka in Mymensingh City, Bangladesh
Source: One Health. 2025 Aug 18;21:101170. doi: 10.1016/j.onehlt.2025.101170 (PMC12396293; doi:10.1016/j.onehlt.2025.101170)
Supplement: Supplementary file 2 — Supplementary material 2 [file mmc2.docx]

**Manuscript tile:**

**Table S1:** Sampling details

| **Categories** | **Shop** | **Sample details** | **Total Sample** | **Grand total Sample** |
| --- | --- | --- | --- | --- |
| Street food vendor | A | Fuchka, salad, hand wash, dish wash | 4 | 20 |
|  | B | Fuchka, salad, hand wash, dish wash | 4 |  |
|  | C | Fuchka, salad, hand wash, dish wash | 4 |  |
|  | D | Fuchka, salad, hand wash, dish wash | 4 |  |
|  | E | Fuchka, salad, hand wash, dish wash | 4 |  |
| Fuchka shop | A | Fuchka, salad, hand wash, dish wash | 4 | 20 |
|  | B | Fuchka, salad, hand wash, dish wash | 4 |  |
|  | C | Fuchka, salad, hand wash, dish wash | 4 |  |
|  | D | Fuchka, salad, hand wash, dish wash | 4 |  |
|  | E | Fuchka, salad, hand wash, dish wash | 4 |  |
| Restaurant | A | Fuchka, salad, hand wash, dish wash | 4 | 20 |
|  | B | Fuchka, salad, hand wash, dish wash | 4 |  |
|  | C | Fuchka, salad, hand wash, dish wash | 4 |  |
|  | D | Fuchka, salad, hand wash, dish wash | 4 |  |
|  | E | Fuchka, salad, hand wash, dish wash | 4 |  |
|  | | |  | **60** |

**Table S2**: Microbial load assessment through determination of total viable count (TVC) of fuchka and fuchka-related samples

| **Category** | **Selling point No.** | **Fuchka** | **Salad** | **Hand wash** | **Dish wash** |
| --- | --- | --- | --- | --- | --- |
|  |  | **CFU/g** | **CFU/g** | **CFU/ml** | **CFU/ml** |
| **Shop based** | 1 | 1.5x10^15^ | 4.42x10^7^ | 2.05x10^9^ | 5.87x10^5^ |
|  | 2 | 7.05x10^7^ | 4.42x10^8^ | 3.12x10^5^ | 7.32x10^7^ |
|  | 3 | ODL | ODL | 1.57x10^5^ | 2x10^5^ |
|  | 4 | 1.6x10^6^ | 4.42x10^10^ | 1.6x10^6^ | 2.64x10^6^ |
|  | 5 | 1.7x10^7^ | 4.42x10^11^ | 1.7x10^8^ | 9.9x10^7^ |
| **Vendor** | 1 | 8.25x10^6^ | 3.75x10^7^ | 5.5x10^7^ | 5.25x10^6^ |
|  | 2 | 7.5x10^4^ | 4.85x10^5^ | 8.9x10^7^ | 6.75x10^6^ |
|  | 3 | 7.5x10^5^ | 7.5x10^6^ | 3.25x10^6^ | 2.62x10^7^ |
|  | 4 | 2.31x10^7^ | 6.6x10^7^ | 3.96x10^6^ | 5.6x10^6^ |
|  | 5 | 3.96x10^4^ | 4.95x10^4^ | 1.32x10^6^ | 9.57x10^5^ |
| **Restaurant** | 1 | 6.75x10^13^ | 7.2x10^14^ | 6.25x10^5^ | 6.25x10^5^ |
|  | 2 | 4.12x10^5^ | 2.12x10^5^ | BDL | 3.37x10^5^ |
|  | 3 | 2.1x10^5^ | 1.27x10^9^ | 1.45x10^9^ | 2.25x10^8^ |
|  | 4 | 4.29x10^4^ | 3.30 x10^4^ | 3.63 x10^4^ | 4.62x10^4^ |
|  | 5 | 5.28 x10^5^ | 6.27 x10^5^ | BDL | 5.28 x10^4^ |

**Note:** CFU= colony forming unit; ‘ODL’ = over detection limit, ‘BDL’ = below detection limit

**Table S3:** Microbial load assessment through determination of total coliform count (TCC) of fuchka and fuchka-related samples

| **Category** | **Selling point No.** | **Fuchka** | **Salad** | **Hand wash** | **Dish wash** |
| --- | --- | --- | --- | --- | --- |
|  |  | **CFU/gm** | **CFU/gm** | **CFU/ml** | **CFU/ml** |
| **Shop based** | 1 | 4.45x10^6^ | 7.75x10^8^ | 3.7x10^5^ | 5.87x10^5^ |
|  | 2 | 1.3x10^7^ | BDL | 7.75x10^4^ | 1.7x10^5^ |
|  | 3 | BDL | 4.48x10^5^ | BDL | BDL |
|  | 4 | 2.3x10^5^ | 6.8x10^4^ | 4.3x10^4^ | 3.3x10^5^ |
|  | 5 | 5.9x10^6^ | 6.6x10^5^ | 9.9x10^7^ | 3.3x10^7^ |
| **Vendor** | 1 | 9.5x10^4^ | 1.97x10^5^ | BDL | BDL |
|  | 2 | BDL | 8.0x104 | 1x10^7^ | 3.32x10^5^ |
|  | 3 | BDL | 1.27x10^5^ | 1.75x10^5^ | 7.5x10^4^ |
|  | 4 | 9.57x10^4^ | 6.6x10^5^ | 3.13x10^6^ | 1.98x10^6^ |
|  | 5 | BDL | BDL | 6.93x10^5^ | 5.61x10^4^ |
| **Restaurant** | 1 | BDL | BDL | BDL | BDL |
|  | 2 | BDL | BDL | BDL | 2.12x10^5^ |
|  | 3 | BDL | ∞ | BDL | 1.08x10^7^ |
|  | 4 | BDL | 9.9 x10^3^ | BDL | BDL |
|  | 5 | 2.31x10^4^ | 1.32 x10^4^ | BDL | BDL |

**Note:** CFU= colony forming unit; ‘ODL’ = over detection limit, ‘BDL’ = below detection limit

**Table S4:** Microbial load assessment through determination of total staphylococcal count (TSC) of fuchka and fuchka-related samples

| **Category** | **Selling point No.** | **Fuchka** | **Salad** | **Hand wash** | **Dish wash** |
| --- | --- | --- | --- | --- | --- |
|  |  | **CFU/gm** | **CFU/gm** | **CFU/ml** | **CFU/ml** |
| **Shop based** | 1 | 1.3x10^5^ | 6.0x10^4^ | 7.75x10^5^ | 2.00x10^4^ |
|  | 2 | 1.82x10^6^ | 1.05x10^5^ | 3.45x10^5^ | 4.17x10^7^ |
|  | 3 | 5.5x10^8^ | 5.65x10^5^ | BDL | 1.75x10^5^ |
|  | 4 | 8.0x10^4^ | 4.4x10^4^ | 6.3x10^5^ | 9.9x10^5^ |
|  | 5 | 9.9x10^4^ | 1.9x10^6^ | 3.9x10^6^ | 9.9x10^4^ |
| **Vendor** | 1 | 6.0x10^5^ | 1.27x10^5^ | BDL | 3.95x10^5^ |
|  | 2 | BDL | 2.32x10^5^ | 9.25x10^9^ | BDL |
|  | 3 | 5.5x10^4^ | 9.25x10^4^ | 1.02x10^5^ | 8.5x10^4^ |
|  | 4 | 6.6x10^3^ | 6.6x10^5^ | 4.29x10^4^ | 1.32x10^4^ |
|  | 5 | BDL | 9.9x10^3^ | 3.96x10^4^ | 9.57x10^4^ |
| **Restaurant** | 1 | 1x10^7^ | 5.5x10^8^ | BDL | 1.75x10^5^ |
|  | 2 | 1.12x10^5^ | BDL | BDL | BDL |
|  | 3 | 9.5x10^4^ | 6.4x10^7^ | 9x10^4^ | 9x10^4^ |
|  | 4 | 1.98x10^4^ | BDL | BDL | BDL |
|  | 5 | BDL | 2.97 x10^4^ | BDL | 1.32 x10^4^ |

**Note:** CFU= colony forming unit; ‘BDL’ = below detection limit

**Table S5:** Selling point category and sample- wise mean and standard deviation of log CFU value of TVC, TCC, TSC

| **Category** |  | **TVC (mean log CFU)** | | | | **TCC (mean log CFU)** | | | | **TSC (mean log CFU)** | | | |
| --- | --- | --- | --- | --- | --- | --- | --- | --- | --- | --- | --- | --- | --- |
|  |  | **Fuchka** | **Salad** | **Hand wash** | **Dish wash** | **Fuchka** | **Salad** | **Hand wash** | **Dish wash** | **Fuchka** | **Salad** | **Hand wash** | **Dish wash** |
| **Shop based** | 1 | 15.176 | 7.645 | 9.311 | 5.768 | 6.648 | 8.889 | 5.568 | 5.903 | 5.113 | 4.778 | 4.301 | 4.301 |
|  | 2 | 7.848 | 7.301 | 5.494 | 7.864 | 7.113 | BDL | 4.889 | 5.230 | 6.260 | 5.021 | 5.537 | 7.620 |
|  | 3 | ODL | ODL | 5.195 | 5.301 | BDL | 5.651 | BDL | BDL | 8.740 | 5.752 | BDL | 5.243 |
|  | 4 | 6.204 | 5.230 | 6.204 | 6.421 | 5.361 | 4.832 | 4.633 | 5.518 | 4.903 | 4.643 | 5.803 | 5.995 |
|  | 5 | 7.230 | 6.965 | 8.230 | 7.995 | 6.770 | 5.819 | 7.995 | 7.518 | 4.995 | 6.278 | 6.591 | 4.995 |
|  | **Mean*** | 10.49 | 8.62 | 6.88 | 6.66 | 5.17 | 5.03 | 4.61 | 4.83 | 6.0 | 5.29 | 4.44 | 5.63 |
|  | **SD*** | 4.69 | 4.22 | 1.79 | 1.21 | 2.97 | 3.21 | 2.90 | 2.84 | 1.62 | 0.69 | 2.61 | 1.26 |
| **Vendor** | 1 | 6.916 | 7.574 | 7.740 | 6.720 | 4.977 | 5.294 | BDL | BDL | 5.778 | 5.103 | BDL | 5.596 |
|  | 2 | 4.875 | 5.685 | 7.949 | 6.829 | BDL | 4.903 | 7.000 | 5.521 | BDL | 5.365 | 6.996 | BDL |
|  | 3 | 5.875 | 6.875 | 6.511 | 7.418 | BDL | 5.103 | 5.243 | 4.875 | 4.740 | 4.966 | 5.009 | 4.929 |
|  | 4 | 7.363 | 7.819 | 6.597 | 6.784 | 4.980 | 5.819 | 6.495 | 6.296 | 3.819 | 5.995 | 4.632 | 4.120 |
|  | 5 | 4.597 | 4.694 | 6.120 | 5.980 | BDL | BDL | 5.840 | 4.748 | BDL | 3.995 | 4.597 | 4.980 |
|  | **Mean*** | 5.92 | 6.52 | 6.98 | 6.74 | 1.99 | 4.22 | 4.91 | 4.22 | 2.86 | 5.08 | 4.24 | 3.92 |
|  | **SD*** | 1.21 | 1.31 | 0.80 | 0.51 | 2.72 | 2.38 | 2.82 | 2.47 | 2.70 | 0.72 | 2.57 | 2.25 |
| **Restaurant** | 1 | 13.829 | 14.860 | 5.439 | 5.795 | BDL | BDL | BDL | BDL | 7.000 | 8.740 | BDL | 5.243 |
|  | 2 | 5.614 | 5.326 | BDL | 5.527 | BDL | BDL | BDL | 5.326 | 5.049 | BDL | BDL | BDL |
|  | 3 | 5.322 | 9.103 | 9.161 | 8.352 | BDL | ODL | BDL | 7.033 | 4.977 | 7.806 | 4.954 | 4.954 |
|  | 4 | 4.632 | 4.518 | 4.559 | 4.664 | BDL | 3.995 | BDL | BDL | 4.296 | BDL | BDL | BDL |
|  | 5 | 5.722 | 5.797 | BDL | 4.722 | 4.363 | 4. 120 | BDL | BDL | BDL | 4.120 | BDL | 4.120 |
|  | **Mean*** | 7.02 | 7.92 | 3.83 | 5.81 | 0.87 | 4.82 | 0 | 2.47 | 4.26 | 4.13 | 0.99 | 2.86 |
|  | **SD*** | 3.82 | 4.25 | 3.90 | 1.50 | 1.95 | 6.52 | 0 | 3.43 | 2.58 | 4.14 | 2.22 | 2.64 |
| **Mean**** | | 6.74 | 7.69 | 5.90 | 6.40 | 2.34 | 4.69 | 3.17 | 3.86 | 4.37 | 4.88 | 3.22 | 4.13 |
| **SD**** | | 3.65 | 3.40 | 2.78 | 1.15 | 3.05 | 4.12 | 3.18 | 2.92 | 2.33 | 2.42 | 2.81 | 2.30 |

**Note:** CFU= Colony forming unit; ‘ODL’ = over detection limit, ‘BDL’ = below detection limit; SD= Standard deviation. ‘*’= selling category wise, ‘**’= sample wise. For statistical analysis (calculating mean value), values exceeding the detection limit were assigned a value of 16 log CFU (as the highest observed value within detection range was 15.17 log CFU/g), and values below the detection limit were assigned a value of 0 log CFU ( as some samples showed no detectable coliforms). These substitutions were made to calculate the mean and standard deviation of TCV (Total viable count), TCC (total coliform count), TSC (Total Staphylococcus count).

**Table S6**: Cultural and Gram-staining properties of isolated bacteria

| **Suspected isolated bacteria** | **Name of media** | **Cultural characteristics** | **Staining Characteristics** | | |
| --- | --- | --- | --- | --- | --- |
|  |  |  | Shape | Arrangement | Gram’s staining character |
| *Escherichia coli* | EMB agar | Deep purple colony with metallic sheen | Short plump rods | Single, paired or in short chain | (-) ve |
| *Klebsiella pneumoniae* | MacConkey agar | Large, mucoid, bright pink, lactose fermented colony | Rod shape | Single, pairs or cluster | (-) ve |
|  | HiCrome UTI agar | blue to purple, mucoid |  |  |  |
| *Enterobacter* spp. | EMB agar | Small greenish black with metallic sheen, silver white colony (different types colony observed for different species) | Rod shape | Single or pair | (-) ve |
| *Citrobacter* spp. | XLD agar | Large yellowish brown colony | Rod shape | Single or pair | (-) ve |
|  | SS agar | Black to black centered pinkish colony resembles *Salmonella* spp. |  |  |  |
| *Staphylococcus* spp. | MS agar | White to medium yellowish colony | Cocci in shape | Arranged in grapes like cluster | (+) ve |

Notes: (+)ve = Positive; (-)ve= Negative

**Table S7**: Biochemical characteristics of isolated bacteria from fuchka and fuchka-related samples

| **Suspected isolated bacteria** | Sugar fermentation test | | | | | Other biochemical tests | | | |
| --- | --- | --- | --- | --- | --- | --- | --- | --- | --- |
|  | Dextrose | Maltose | Lactose | Mannitol | Sucrose | MR | VP | Indole | Catalase |
| *Escherichia coli* | + | + | + | + | + | + | - | + | + |
| *Klebsiella pneumoniae* | + | + | + | + | + | - | + | - | + |
| *Enterobacter* spp. | + | + | - | + | + | - | + | - | + |
| *Citrobacter* spp. | + | + | - | + | - | + | - | - | + |
| *Staphylococcus* spp. | + | + | + | + | + | + | + | - | + |

**Legends**: MR: Methyl Red; VP: Voges-Proskauer; +: positive; -: negative

**Table S8**: Bacterial isolates positively identified using the MALDI Biotyper technique

| **Sl. No.** | **Isolate Number** | **Score value** | **MALDI-TOF MS Analysis** |
| --- | --- | --- | --- |
|  |  |  | **Best Match** |
| **1** | 1(1) | 2.22 | *Enterobacter kobei* |
| **2** | 1(3) | 2.30 | *Staphylococcus warneri* |
| **3** | 1(4) | 2.16 | *Enterobacter bugendensis* |
| **4** | 2(1) | 1.84 | *Pontoea stewartii* |
| **5** | 2(2) | 2.10 | *Enterobacter kobei* |
| **6** | 2(3) | 2.16 | *Escherichia coli* |
| **7** | 3(1) | 2.14 | *Staphylococcus hominis* |
| **8** | 3(2) | 2.33 | *Escherichia coli* |
| **9** | 3(3) | 2.15 | *Enterobacter asburiae* |
| **10** | 3(4) | 2.24 | *Citrobacter freundii* |
| **11** | 3(5) | 2.31 | *Escherichia coli* |
| **12** | 3(8) | 2.02 | *Escherichia coli* |
| **13** | 3(9) | 2.26 | *Escherichia coli* |
| **14** | 3(10) | 2.28 | *Escherichia coli* |
| **15** | 3(11) | 2.24 | *Escherichia coli* |
| **16** | 3(13) | 2.23 | *Enterobacter bugendensis* |
| **17** | 3(14) | 2.21 | *Escherichia coli* |
| **18** | 3(15) | 2.23 | *Enterobacter cloacae* |
| **19** | 3(16) | 2.11 | *Citrobacter freundii* |
| **20** | 3(18) | 2.29 | *Escherichia coli* |
| **21** | 3(20) | 2.29 | *Escherichia coli* |
| **22** | 4(1) | 1.77 | *Cronobacter* spp. |
| **23** | 4(2) | 2.32 | *Serratia marcescens* |
| **24** | 4(5) | 2.23 | *Serratia marcescens* |
| **25** | 5(3) | 2.35 | *Citrobacter freundii* |
| **26** | 5(4) | 2.02 | *Raoultella ornitholytica* |
| **27** | 5(6) | 2.25 | *Enterobacter cloacae* |
| **28** | 5(7) | 2.10 | *Enterobacter cloacae* |
| **30** | 5(8) | 2.32 | *Leclercia adecarboxylata* |
| **31** | 5(9) | 2.09 | *Raoultella ornitholytica* |
| **32** | 5(10) | 2.29 | *Citrobacter freundii* |
| **33** | 5(12) | 2.21 | *Enterobacter kobei* |
| **34** | 5(13) | 2.13 | *Enterobacter kobei* |
| **35** | 7(1) | 1.75 | *Enterobacter cloacae* |
| **36** | 7(2) | 2.30 | *Enterobacter bugendensis* |
| **37** | 7(4) | 2.11 | *Areomonas caviae* |
| **38** | 7(5) | 2.29 | *Enterobacter kobei* |
| **39** | 8(1) | 2.16 | *Escherichia coli* |
| **40** | 8(2) | 2.39 | *Klebsiella pneumoniae* |
|  | | | |

**Table S8**: Bacterial isolates positively identified using the MALDI Biotyper technique (continued)

| **Sl. No.** | **Isolate Number** | **Score value** | **MALDI-TOF MS Analysis** |
| --- | --- | --- | --- |
|  |  |  | **Best Match** |
| **41** | 8(3) | 2.21 | *Acinetobacter baumannii* |
| **42** | 8(4) | 2.27 | *Klebsiella pneumoniae* |
| **43** | 8(5) | 2.25 | *Klebsiella pneumoniae* |
| **44** | 8(6) | 2.21 | *Klebsiella pneumoniae* |
| **45** | 8(8) | 2.02 | *Enterobacter kobei* |
| **46** | 8(9) | 2.29 | *Citrobacter freundii* |
| **47** | 8(11) | 2.26 | *Enterobacter bugendensis* |
| **48** | 8(12) | 1.98 | *Staphylococcus warneri* |
| **49** | 8(13) | 2.12 | *Staphylococcus warneri* |
| **50** | 8(15ii) | 2.02 | *Enterobacter bugendensis* |
| **51** | 8(15iii) | 2.35 | *Klebsiella pneumoniae* |
| **52** | 9(3) | 2.02 | *Staphylococcus hominis* |
| **53** | 9(7) | 2.33 | *Klebsiella pneumoniae* |
| **54** | 9(9) | 1.73 | *Pontoea agglomerans* |
| **55** | 9(10) | 2.27 | *Citrobacter freundii* |
| **56** | 9(11) | 2.20 | *Enterobacter bugendensis* |
| **57** | 9(12) | 2.28 | *Citrobacter freundii* |
| **58** | 9(13) | 2.26 | *Citrobacter freundii* |
| **59** | 9(14) | 2.33 | *Citrobacter freundii* |
| **60** | 9(15) | 2.20 | *Citrobacter freundii* |
| **61** | 9(16) | 2.31 | *Citrobacter freundii* |

**Table S9:** Antibiogram result of Gram-negative bacteria isolated from fuchka samples

| **Name of Antibiotic** | **Sensitivity pattern** | **Bacterial genera** | | | |
| --- | --- | --- | --- | --- | --- |
|  |  | *E. coli* (n=21) | *Klebsiella pneumoniae* (n=30) | *Enterobacter* spp. (n=23) | *Citrobacter freundii* (n=14) |
| Amoxicillin | Sensitive | 0 | 0 | 0 | 0 |
|  | Intermediate | 1 (4.76%) | 0 | 0 | 0 |
|  | Resistance | 20 (95.24%) | 30 (100%) | 23 (100%) | 14 (100%) |
| Azithromycin | Sensitive | 0 | 0 | 1 (4.76%) | 6 (42.9%) |
|  | Intermediate | 0 | 0 | 14 (60%) | 4 (28.6%) |
|  | Resistance | 21 (100%) | 31 (100%) | 8 (34.8%) | 4 (28.6%) |
| Aztreonam | Sensitive | 19 (90.47%) | 30 (96.67%) | 23 (100%) | 14 (100%) |
|  | Intermediate | 1 (4.76%) | 0 | 0 | 0 |
|  | Resistance | 1 (4.76%) | 1 (3.33%) | 0 | 0 |
| Ceftriaxone | Sensitive | 4 (19.04%) | 10 (33.3%) | 12 (52.1%) | 14 (100%) |
|  | Intermediate | 7 (33.3%) | 12 (40%) | 9 (39.1%) | 0 |
|  | Resistance | 10 (47.62%) | 8 (26.67%) | 2 (8.7%) | 0 |
| Levofloxacin | Sensitive | 20 (95.24%) | 30 (96.67%) | 23 (100%) | 13 (93%) |
|  | Intermediate | 1 (4.76%) | 0 | 0 | 1 (7%) |
|  | Resistance | 0 | 1 (3.33%) | 0 | 0 |
| Gentamicin | Sensitive | 21 (100%) | 23 (76.67%) | 0 | 5(35%) |
|  | Intermediate | 0 | 4 (13.33%) | 15 (65.2%) | 8 (57%) |
|  | Resistance | 0 | 3 (10%) | 8 (34.8%) | 1 (7%) |
| Nalidixic Acid | Sensitive | 0 | 16 (53.3%) | 21 (91.3%) | 12 (86%) |
|  | Intermediate | 12 (57.14%) | 9 (30%) | 2 (8.7%) | 2 (14.3%) |
|  | Resistance | 9 (42.85%) | 5 (16.67%) | 0 | 0 |
| Meropenem | Sensitive | 16 (76.19%) | 25 (83.33%) | 23 (100%) | 14 (100%) |
|  | Intermediate | 5 (23.81%) | 5 (16.67%) | 0 | 0 |
|  | Resistance | 0 | 0 | 0 | 0 |
| Oxytetracycline | Sensitive | 14 (66.7%) | 22 (73.3%) | 23 (100%) | 14 (100%) |
|  | Intermediate | 3 (14.3%) | 0 | 0 | 0 |
|  | Resistance | 4 (19%) | 8 (26.7%) | 0 | 0 |
| Co-trimoxazole | Sensitive | 21 (100%) | 30 (100%) | 23 (100%) | 14 (100%) |
|  | Intermediate | 0 | 0 | 0 | 0 |
|  | Resistance | 0 | 0 | 0 | 0 |

**Table S10:** Antibiogram result of Gram-positive bacteria (*Staphylococcus* spp.) isolated from fuchka samples

| **Bacterial genera (*Staphylococcus* spp.)** | **Sensitivity pattern** | **Name of Antibiotic** | | | | | | | | | |
| --- | --- | --- | --- | --- | --- | --- | --- | --- | --- | --- | --- |
|  |  | AMX | TE | CIP | CTR | VA | COT | CN | LNZ | C | E |
|  | Sensitive | 0 | 24 (92.3%) | 22 (84.6%) | 2 (7.7%) | 26 (100%) | 26 (100%) | 21 (80.8%) | 26 (100%) | 25 (96.1%) | 9 (34.6%) |
|  | Intermediate | 0 | 1 (3.8%) | 2 (7.7%) | 8 (30.8%) | 0 | 0 | 3 (11.5%) | 0 | 0 | 3 (11.5%) |
|  | Resistance | 26 (100%) | 1 (3.8%) | 2 (7.7%) | 16 (61.5%) | 0 | 0 | 2(7.7%) | 0 | 1 (3.8%) | 14 (53.8%) |

**Note:** AMX = amoxicillin, TE = tetracycline, CIP = ciprofloxacin, CTR = ceftriaxone, VA = vancomycin, COT = cotrimoxazole, CN = gentamicin, LNZ = linezolid, C = chloramphenicol, E = erythromycin.

**Table S11**: Comparison of microbiological findings in fuchka and similar street foods from selected regions of Bangladesh

| **City/Study** | **TVC (CFU/g)/**  **log mean CFU** ± **SD** | **TCC (CFU/g)** | **TSC (CFU/g)** | **MDR Bacteria Identified** | **Reference** |
| --- | --- | --- | --- | --- | --- |
| Current Study | 5.2 × 10⁵ | 3.8 × 10⁴ | 1.6 × 10⁴ | *E. coli*, *S. aureus*, *K. pneumoniae* | [This study] |
| Dhaka | 8×10^7^ | 7.9x10^6^ | 1.9 × 10⁴ | *E. coli*, *Vibrio cholerae* | [18] |
| Dinajpur | 2.5 × 10^4^ | - | - | *E. coli*, *K*. *pneumoniae, Salmonella* | [7] |
| Mymensingh | 5.35 ± 0.59 | 4.10±0.73 | 4.47 ± 0.61 | *E. coli*, *Staphlococcus* spp. | [2] |
| Tangail | 8.9 ×10^7^ | 7.1×10^7^ | 4.6×10^6^ | *E. coli, S. aureus.* | [26] |
